# Evaluation of a peer-support, ‘mentor mother’ program in Gaza, Mozambique; a qualitative study

**DOI:** 10.1186/s12913-024-10833-3

**Published:** 2024-03-27

**Authors:** Leila Katirayi, Sozinho Ndima, Amgad Farah, Whitney Ludwig, Atanásio Mabote, Ismail Chiposse, Ana Muteerwa, Jessica Greenberg Cowan, Ivete Meque

**Affiliations:** 1 Research Department, Elizabeth Glaser Pediatric AIDS Foundation, Washington, DC USA; 2 Research Department, Elizabeth Glaser Pediatric AIDS Foundation, Maputo, Mozambique; 3 Milken Institute School of Public Health, Department of Prevention and Community Health, George Washington University, Washington, DC USA; 4 Milken Institute School of Public Health, Department of Public Health, George Washington University, Washington, DC USA; 5 XX Department, Elizabeth Glaser Pediatric AIDS Foundation, Maputo, Mozambique; 6 Ministry of Health, Provincial Director for Gaza, Gaza, Mozambique; 7 Division of Global HIV & TB, Centers for Disease Control and Prevention, Maputo, Mozambique

**Keywords:** HIV, Peer support, Mentor mothers, PMTCT, Mozambique, HIV-positive mothers

## Abstract

**Background:**

Retention in prevention of mother-to-child transmission of HIV programs is critical to reduce vertical transmission. To addresses challenges with retention, Mozambique launched a peer-support program in 2018, in which HIV-positive mothers provide adherence support as mentor mothers (MMs) for HIV-positive pregnant and lactating women and HIV-exposed and infected children.

**Methods:**

A descriptive qualitative evaluation was conducted across nine facilities in Gaza Province to assess the acceptability and barriers to implementation of the mentor mother program (MMP) among those receiving services and providing services. In-depth interviews and focus group discussions were conducted with MMs, MM supervisors, health care workers (HCWs), HIV-positive mothers enrolled in the MMP, HIV-positive mothers who declined MMP enrollment, and key informants involved in the implementation of the program. Thematic analysis identified emerging recurrent themes and patterns across the participants’ responses. Data were collected between November-December 2020.

**Results:**

There were initial challenges with acceptability of the MMP, especially regarding confidentiality concerns and MM roles. Sharing additional information about MMs and making small changes during the beginning of the MMP resulted in generally high acceptance of the MMP. HIV-positive mothers reported that counseling from MMs improved their understanding of the importance of anti-retroviral treatment (ART) and how to take and administer ART. HIV-positive mothers reported having reduced guilt and shame about their HIV-status, feeling less alone, and having more control over their health. MMs shared that their work made them feel valued and decreased their self-stigmatization. However, MMs also reported feeling that they had inadequate resources to perform optimal job functions; they listed inadequate transportation, insufficient stipends, and false addresses of clients among their constraints. Overall, HCWs felt that their workload was significantly reduced with MM support and wanted more MMs in the community and health facility.

**Conclusions:**

This study found that the MMP was considered a substantive and highly valued support to HIV-positive mothers, resulting in increased ART literacy among patients, improved self-reported well-being and sense of community and reduced feelings of isolation. Recommendations include strengthening MM training, increasing financial and materiel resources, additional information provided to newly enrolled mothers and support for the male partners.

**Supplementary Information:**

The online version contains supplementary material available at 10.1186/s12913-024-10833-3.

## Background

In the past decade, significant progress has been made in the prevention of mother-to-child transmission (PMTCT) of HIV [1, 2]. Nevertheless, each year, more than 700,000 pregnant women and women of reproductive age contract HIV worldwide. More than 70% of these women live in just 23 countries, most of which are in sub-Saharan Africa [1, 3]. In the absence of treatment, the risk of mother-to-child transmission of HIV during pregnancy, childbirth, and breastfeeding can be as high as 45% [1]. In Mozambique, approximately 150,000 children ages 0–14 are living with HIV [4], the national HIV prevalence is 12.4% % among adults aged 15–49 and the country’s highest prevalence is Gaza Province at 20.9% [5]. At the time the national mentor mother strategy was launched in 2018, the government of Mozambique reported that retention rates in HIV care and treatment among pregnant women were 65% in Gaza Province versus 81% nationally [6]. Strengthening retention among HIV-positive pregnant and lactating women is a priority both because improved retention improves the health of women, and it reduces vertical transmission and infant morbidity and mortality.

## Challenges with PMTCT

Poor retention of mothers and children in care, inadequate social support, transportation costs, and stigma have been identified as barriers that impede the success of PMTCT strategies [7]. Delays at the facilities, overcrowding, long wait times, and privacy concerns during counseling sessions result in women discontinuing PMTCT interventions. The success of PMTCT interventions may also be hindered by poor motivation among pregnant and lactating women and health care workers (HCWs), inadequate support systems, and low adherence to medication and appointments [7–9].

### Mentor mother program - a peer-led approach to HIV programming

One potential approach to overcoming challenges to PMTCT programs is by providing peer support, such as Mentor Mothers (MMs). MMs are HIV-positive mothers who have completed the PMTCT cascade, the clinical program intended to prevent HIV transmission during pregnancy, labor, delivery, and breastfeeding [7–12]. The MM model strengthens the PMTCT cascade by optimizing HCWs’ time by shifting tasks from the HCWs to the MMs and empowering individuals living with HIV to support one another via a formal mentoring and counseling strategy. The MMs provide psychosocial support and education to HIV-positive pregnant and lactating mothers and serve as role models, thereby promoting retention [13].

Mentor Mothers are trained to provide group and one-on-one support to HIV-positive pregnant and lactating women. They encourage enrollment, adherence, and retention in HIV care and treatment and share their own experiences as HIV-positive mothers. Their scope of work includes tracing women who miss their clinic visits via phone calls and home visits and counseling HIV-positive mothers on PMTCT [14, 15]. By 2017, MM programs (MMPs) had supported over two million women living with HIV in sub-Saharan Africa in countries such as South Africa, Uganda, Ethiopia, Malawi, and Nigeria. The programs have resulted in reported decreases in mother-to-child transmission rates, increased ART adherence, improved uptake of early infant HIV diagnosis, and reduced workloads for HCWs [14].

Igumbor et al. used secondary data obtained from a Uganda MMP to assess the influence of MMs on the retention of mother-infant pairs in HIV-care by comparing mother-infant pairs attending the standard care of PMTCT services to the PMTCT services received through the MMP [7]. They found an increase in the number of mother-baby pairs retained at six weeks after birth (96.7% versus 65.8%), six weeks after cessation of breastfeeding (81.5%versus 42%), and 18 months after birth (71.2% versus 20.6%) when supported by the MMs [7].

## Mentor mother program in Mozambique

In 2016, the Elizabeth Glaser Pediatric AIDS Foundation (EGPAF), in collaboration with the Mozambique Ministry of Health launched the MMP (originally called “Educator Mothers”) in Gaza province. The MMP was launched in 100 high-volume facilities with high-HIV prevalence, including health centers and rural hospitals. The program trained HIV-positive mothers to provide support as MMs who then conducted home visits for HIV-positive pregnant and lactating women and HIV-exposed and infected children. To be eligible to be a MM, the HIV-positive mothers had to have been compliant in care and treatment. The introductory five-day training included guidance on conducting home visits, adherence counseling, screening for tuberculosis and malnutrition, screening for other sexual transmissible infections, completing program forms, and additional topics regarding pregnancy and the postpartum period. Depending on their individual performance during the training, MMs were assigned to one of the following positions: MM focal point, MM supervisor, or MM. The MM focal points were based at health facilities and were responsible for the coordination of the MMP, demand creation, and follow-up for referrals. The MM supervisors performed home visits and provided technical support and supervision for a maximum of 10 MMs and MMs only performed the home visits.

### Study aim

Previous research has found that MMPs are associated with improved retention in prevention of vertical transmission services and higher viral suppression rates among pregnant and postpartum women [16]. However, initial feedback from EGPAF program staff at the facilities reported challenges with the MMP program, including women not accepting MM visits in their homes, MMs facing challenges with male partners during home visits, HCWs not accepting the MMs at the facility, confidentiality concerns and lack of sufficient stipends for MMs. This study was conducted to better understand the challenges of implementing the MMP program, and how these challenges can be addressed. This paper explores the acceptability of the MMP, barriers to implementation and provides recommendations for improvement.

## Methods

### Study design

A qualitative descriptive study [17] was conducted using data collected through in-depth interviews (IDIs) and focus group discussions (FGDs).

### Study population

The study population was comprised of MMs, MM supervisors, HCWs from the antenatal care and child at-risk units, HIV-positive mothers enrolled in the MMP, HIV-positive mothers who refused MMP enrollment, and key informants including MMP managers, MMP coordinators, and district mother focal points.

Eligibility criteria for MMs and MM supervisors to participate in the study required that they had provided services in their role in the selected sites for at least six months in the previous year. Eligible HCWs were nurses who worked in antenatal care and child at-risk units at the health facilities where the study took place for at least six months in the previous year. Eligible HIV-positive mothers were 18 years of age, pregnant or postpartum and had been invited to join the MMP; subjects included (study those who accepted and those who declined participation). Eligible Key informants were EGPAF staff, responsible for the MMP design, implementation, and monitoring. All participants were at least 18 years old. See Table 1 for an overview of data collected.

**Table 1:** Overview of data collected by study population

Population		IDI	FGD
**MMs**			8
**MM supervisors**		11	
**HCWs**		15	
**HIV-positive mothers**	Enrolled in the MMP	45	
	Refused to enroll in the MMP	9	
**Key informants**	MMP manager	1	
	MMP coordinator	2	
	MM focal point	9	
**TOTAL**		**92**	**8**

### Site selection

The MMP was implemented by EGPAF in 12 of the 14 districts in Gaza province (the second most southern province of Mozambique, bordering South Africa and Zimbabwe). Nine health facilities were selected for the study based on the presence of a focal point for the MMP. Seven sites were health centers located in Bilene, Xai-Xai, Chongoene, Chibuto, Guijá, Chockwé, and Mabalane, and two rural hospitals were selected in Manjakazi and Chicumbane.

### Data collection

EGPAF hired short-term staff located in Gaza called Evaluation Assistants (EAs), to collect the data. The EAs had research experience and held undergraduate degrees in social sciences. They were from Gaza province and familiar with the local culture. The EAs had no prior relationship to the MMP or the study. EAs introduced themselves and the study objectives to all study participants prior to obtaining informed consent.

Data were collected between November- December 2020. EAs conducted IDIs and FGDs using semi-structured guides designed for this study (see Appendix A-G in the supplementary material). Because data collection took place during the peak of the COVID-19 pandemic, the data collection guides were not piloted. Instead, the data collection training had an extended role-playing section for the EAs to become intimately familiar with the guides.

IDIs lasted 30–40 min on average, while FGDs lasted approximately one hour and twenty minutes. EAs were instructed to take notes regarding all non-verbal communications or behaviors (referred to as ‘field notes.’) All interviews and FGDs were conducted at the health facilities in private rooms where conversations could not be heard. All interviews were completed and no repeat interviews were needed. All IDIs and FGDs were audio-recorded.

The guides were designed in English and translated into Portuguese and Changana, which is one local language in Southern Mozambique. These data collection tools were back-translated into participants’ languages by those fluent in Portuguese and Changana to ensure accuracy and comprehensibility. Demographic data for the IDI and FGD participants were also collected. FGDs were comprised of 5–12 participants, led by a moderator RA and assisted by a notetaker RA. Individual interviews consisted of one participant and one interviewer. The tools asked about experiences with the MMP since its inception in 2016. See supplemental material for guides. Audio-recordings were transcribed by the EAs and translated from Changana to Portuguese and then to English. A total of 92 IDIs and eight FGDs were conducted with respective groups. Please see Table 1 for an overview of data collected.

### Sampling

For each of the nine sites, we aimed to conduct one FGD with MMs and interview one MM supervisor, two HCWs, up to five HIV-positive mothers and one HIV-positive mother who did not enroll. We aimed to interview all eligible key informants. Sample sizes were based on previous literature that indicated that saturation is reached at 10–12 interviews and 3–6 FGDs per a homogenous group [18, 19].

HIV-positive mothers were divided into two subgroups, those who enrolled in the MMP and those who did not. Three to five mothers enrolled in the program were selected using simple random sampling from a list of eligible HIV-positive mothers. EAs obtained a list of those who did not accept participation in the MMP from HCWs, and they were contacted in the order of most recent refusal. All of the HIV-positive mothers were contacted over the phone by health facility staff. The interviews were set on a day that the HIV-positive mother was already scheduled to return to the health facility for an appointment to avoid any inconvenience.

EAs randomly selected a MM and an MM supervisor from each district to participate by placing eligible MM and MM supervisors’ names in a bag. If any selected individuals refused to participate, additional participants were selected. The MMs and MM supervisors were contacted on the phone by the MM District Supervisors. Similarly, EAs asked the head nurse or the HCW in-charge of the facility in each district to provide a list of all eligible HCWs who were working during the weeks of data collection. Once this list was obtained, HCWs for each district were randomly selected by the EAs. All eligible key informants working in the program were invited to participate in the study. All health facility staff (including MM supervisors) were contacted by the head nurse in person or over the phone to schedule the interview when they were already scheduled to be at the health facility. All recruited participants agreed to participate. Reporting for this study adheres to the consolidated criteria for reporting qualitative research (COREQ) guidelines.

### Analysis

Thematic analysis was employed using a combined inductive and deductive approach. Using an inductive approach, two RAs trained in qualitative analysis and one of the principal investigators (the qualitative lead) reviewed transcripts and individually identified potential codes from the data. The RAs and the PI agreed as a group which codes best reflected the data. Codes were naturally grouped into over-arching topic areas, referred to as ‘themes,’ creating the first code list. Next the PI and RAs reviewed all the IDI and FGD guides to see if the code list would cover all of the topics raised in the guides, adding new codes as needed (using a deductive approach). The two RAs trained in qualitative analysis coded the same transcripts, compared the assigned codes for similar text segments, and resolved any discrepancies by refining or introducing new codes. Once the code list was finalized, the two RAs coded the transcripts individually. Data were analyzed by a thematic analysis approach by study population group: HIV-positive mothers who enrolled in MMP, HIV-positive mothers who refused MMP, MM supervisors, key informants and HCWs. Coding was conducted using the qualitative analysis software, MAXQDA, v.18.

Once all transcripts were coded, code reports were generated for each code. Data reduction and summary tables were generated from the code reports. These tables summarized data into descriptive, text-based summaries that also included text excerpts representative of the identified themes to provide a comprehensive overview of the results.

The frequency in which findings were expressed among participants have been summarized in statements such as a couple (two), a few (max of 4 or 35-40%), half (50%), some (60–65%), most, many/majority (80-85%) of the total number. Some statements may have been less common because they were not directly asked about in the data collection tool, but participants felt that the issue was important and raised it. Participants did not provide feedback on the findings.

## Results

The mean age for HIV-positive mothers was approximately 30 years and 62% of those participating in the MMP were married. Across both groups of mothers, all women reported they had disclosed their HIV status to at least one person in their home. At the time of enrollment in the study, mothers who were enrolled in the MMP reported having known their HIV status and been on ART for a median of 72 months, compared to women who did not accept MMP enrollment, who reported having known their status and being on ART for a median of 45 months (Table 2).

**Table 2:** Demographic data for HIV-positive mothers enrolled and refused enrollment in the MMP

	HIV-positive mothers enrolled *N* = 45 (%)	HIV-positive mothers refused enrollment *N* = 9
Age (years)		
Mean [standard deviation]	30.1 [6.19]	30.4 [6.56]
**Marital status**		
Single	16 (36)	2 (2)
Married	28 (62)	7 (8)
**Education level**		
No education	13 (29)	3 (3)
Primary education	18 (40)	2 (2)
Secondary education	14 (31)	4 (4)
**Time knowing HIV status (months)**		
Median (IQR 25, 75)	66 (35.75, 84)	45 (12, 72)
**Time on ART (months)**		
Median (IQR 25, 75)	72 (35.75, 87)	45 (12, 72)

*One HIV-positive enrolled woman was a widow

A total of 70 MMs participated in the eight FGDs. The mean age was 35 years old. All of them had completed some formal education, with 60% (*n* = 42) having secondary education; 76% (*n* = 53) had been an MM for three or more years.

Among the individuals who participated in the IDIs, 11 were MM supervisors with a mean age of 36 years. Eight of the MM supervisors had a secondary education, seven had been a supervisor for at least three years, and one had been in the role for one year. Additionally, 15 HCWs participated in the study, all of whom were female and had been in their position for at least 12 months. The majority of the HCWs were nurses.

A total of 12 key informants were interviewed, which included one MM program manager, two MM program coordinators, and nine MM focal points. Data not shown.

## Results

### Theme 1: acceptability of the mentor mother program

Many MMs reported that at the beginning of MMP implementation they struggled with acceptance from the HIV-positive women and were often treated poorly. One MM Supervisor reported that prior to the MMP many patients had abandoned treatment. HIV-positive mothers feared ART treatment and believed that they had to endure this illness alone. However, perceptions changed with more exposure to the MMP and MMs began to experience fewer difficult cases.

*…while in the past treatment was full of abandonment because there were no mentor mothers who supported this issue. So many people were afraid and abandoned it. So, the advantage is that people go back to the hospital… It is no longer full of people who have defaulted. Because, in the old days, people were afraid of like, hey… this disease is just me… so, now they know that, hey… we all live in it. So, the advantage is this… that I see her, that there are mentor mothers here in the hospital.* (MM Supervisors, 09SM0013)

The MMP came to be generally accepted by the HIV-positive mothers receiving MMs. One of the aspects that contributed significantly to the acceptance of the program was the increased understanding about ART and its importance through messages about how to take ART, how ART protects women and their children and how ART can allow one to live a longer life. HIV-positive mothers also reported that they had a better understanding of why they should return to the health facility and the health benefits they could expect.

*What I really like about this program is to be sensitized, encouraged to take medication, not to drop out of treatment, take medication at the same time every day… It is important because I feel so good in my life when complying with the treatment, and when they (MMs) also help me in giving advice to continue with the medication…* (HIV-positive mother– enrolled, 02MI0008).

HIV-positive mothers enrolled in the MMP reported feeling very comfortable with MMs. This feeling of comfort may have been due to HIV-positive mothers knowing that the MMs had shared similar experiences with their patients. A few HCWs and a key informant stated that HIV-positive mothers felt more at ease speaking with MMs in comparison to health care professionals. An enrolled HIV-positive mother indicated that there are things she might feel too scared to discuss with a HCW, but she would ask questions and talk with the MM during a home visit. Many of the enrolled HIV-positive mothers expressed feeling better and less worried about treatment after MMs shared their experiences.

*I feel good in my heart and I feel good in my body too. While I’m worried about taking the pills… So that I too can live and be able to raise my children…This is where they [MMs] encourage me… I follow the rules, because I know that the [MMs], encourage me and I also realize that life is not bought, so I have to comply with everything they tell me. I also give my heart… Because that person who encourages you considers you as a mother and father would.* (HIV-positive mothers enrolled, 06MI0005)

Many HIV-positive mothers found it helpful to have MMs attend clinical appointments with them, stating that MMs provided moral support and advocated for HIV-positive mothers if conflict arose with the HCWs (such as HCWs being upset with women for not returning earlier). Additionally, HIV-positive mothers presenting with an MM at the health facility were guaranteed to be seen first, saving them time in the queue.

*We sometimes find people who do not accept going to the hospital… if they are afraid we will accompany them… They say they are afraid of the delay in attending the hospital. And we have sensitized them to facilitate their care at the hospital.*. (MM, 04MM0001)

Both key informants and enrolled HIV-positive mothers discussed how MMs alleviate feelings of fear and shame among the HIV-positive mothers; this leads to the HIV-positive mothers feeling empowered to disclose their HIV status to other members of the family. This included MMs assisting with partner counseling and, sometimes, having a male mentor from a program similar to the MMP come to HIV-positive mothers’ homes to talk to their male partners.

*They adapted a way to test us all so that it looks like we’re testing for the first time, so that he [male partner] doesn’t discover that I take pills and so that we learn our HIV status together… But my husband said, hey… I don’t believe it. I’ll go to another hospital in Xin.* (HIV-positive mother– refused enrollment, 05MR0009).

Many MMs shared that their work in the MMP made them feel valued and important, with decreased self-stigmatization towards themselves. MMs explained they experienced feelings of satisfaction due to having an impact on others’ lives and discussed no longer feeling ‘alone’, as the MMP provided a sense of community.

*What I like most is that when I’m with the group I feel good; my heart is not poor. Because when I see the sisters [other HIV-positive mothers], I see myself too, and I feel very happy*. (MM, 08MM0001).

HCWs reported that their workload was significantly reduced with the MMs’ support, and they wanted more MMs in the community and within more units at the facilities. HCWs strongly valued MMs bringing HIV-positive who were lost to follow-up back to the facility. They also noted a that MMs improved HCWs’ insight to their patients’ health conditions.

*…Because they [MMs} help us. They help us, they do a wonderful job, they also reduce what is our burden…* (Key informant, 01CP0001).

### Theme 2: implementation challenges with the MMP

One of the initial challenges was lack of communication around the role of the MMs. MM Supervisors reported that when the MMP was first implemented most HCWs did not understand the purpose of MMs, their duties, or how they could be helpful. Key informants reported that HCWs did not understand how MMs could help facilitate messages regarding medication adherence, the importance of returning to the health facility and other key messages. Initially MMs were excluded from health unit meetings, HCWs refused to see MMP patients, and MMs were called derogatory names. A couple of MM Supervisors stated that there was pushback from HCWs at the beginning of the MMP, but after a few meetings the situation was resolved. HCWs started to appreciate and value the work of the MMs. Once HCWs started to understand how MMs could help them retain HIV-positive mothers as patients, health visits were revised to accommodate a patient’s time with the nurse and the MM.

*…And what was a little challenging for me was because of the rotation of the nurses and to make the nurses understand that the MMs are there to work directly… the nurse did not know what was the role of the mentor mother.* (Key informant, 03PF0002)

It wasn’t only the HCWs that didn’t fully understand the MMP. Some MMs reported being unsure of their roles, how to handle certain situations and generally needing more ongoing support. Individuals who were promoted to a new role in the MMP also reported wanting more transitional training.

*I entered without a clue. I went to training, of course. But I entered without the notion of where and how to start. But I had people there to support me…* (Key informant, 05PF0002).

There were also challenges with HIV-positive mothers understanding the roles of the MMs. Many MMs reported at the onset of the MMP, HIV-positive mothers were fearful and non-trusting of the MMs during their first home visit. HIV-positive mothers were very concerned that the MM might disclose their HIV status to others in the home.

Initially there were concerns about MMs visiting homes, but this was mostly resolved by removing materials that could identify MMs, such as uniforms, bicycles, and folders, and by introducing MMs to community leaders and members to address concerns of ‘strangers’ wandering around neighborhoods.

*What really screwed us up, were the T-shirts…they hate the T-shirts because, the T-shirts, even the neighbor can see that that one is the one she works with, they say that these T-shirts are AIDS…For that reason, they rejected us…* (MM, 03MM0001).

Male partners were also a challenge cited by most respondents because some male partners would reportedly not allow MMs to enter their homes or interact with their wives. Some participants reported that male partners threatened MMs.

*Sometimes the husband does not accept for the mother mentor to visit, and sometimes even with a machete he threatens MMs saying, ‘I’m going to cut you if you don’t want to leave my house’…* (MM supervisor, 07SM0003).

One of the main challenges expressed by the MMs was inadequate resources to do their job, such as insufficient stipends, not enough phone credits to call patients and/or supervisors, and having to use their own resources (such as soap and masks during COVID-19).

… *mentor mothers work a lot, but the subsidy it is not suitable, it should improve a little bit in the subsidy part…* (MM supervisor, 07SM0003).

MMs also felt their workloads were unrealistic and there were not enough MMs to meet the number of women who required support. This was especially problematic when MMs would have to travel long distances in order to reach HIV-positive mothers in peripheral communities.

*Maybe if the MM’s leadership could afford at least a little time for the MMs to rest because as far as I know, the MMs have no right to vacation. Sometimes I think it’s suffocating to be working every day from January to December, from Monday to Friday and a little difficult.* (HCW, 01PS0007)

Many MMs noted challenges locating HIV-positive mothers in the community due to incorrect addresses, not having a phone number, and many of the women working during the day.

*So, this job is very difficult to find people, because addresses are not clear…you go but you just don’t find the home…we don’t know why they give fake addresses…* (MM, 02MM0001).

A number of respondents also reported that they did not have enough space at the facilities to counsel women, which led to counseling sessions in which patients were uncomfortable speaking while other people were in the room.

### Theme 3: recommendations to improve the MM program

HIV-positive mothers enrolling in the MMP requested clearer communication about what happens when an MM makes a home visit and what confidentiality practices are in-place to protect them.

One HCW recommended that MM training be extended for more than a week. A key informant suggested that MMs attend the training provided to new recruits as it would be a refresher and will boost their confidence in supporting HIV-positive mothers. Likewise, continuous training was requested, including refresher sessions, be provided to the MMs as the program continues to evolve.

MMs and HCWs recommended improvements to the MM training and support, including enhanced training to help prepare MMs for their first visit to an HIV-positive woman’s home and more training when entering a new role. Providing an adequate support system to help them handle problems in the field was also suggested.

*… we would like to continue learning*… *renew knowledge and continuing to learn new things that we didn’t learn in the first training. There was a time that we had a training to work with phones…* (MM, 07MM0001).

Among HCWs, MM Supervisors, and key informants, a stronger link with the community was reported as being important for continued MMP implementation. One suggestion was having a community member partner with the MMP to help identify the homes of women. This community member would not need to be informed that the MMP is providing HIV services.

Additionally, HCWs, MMs and HIV-positive mothers recognized that more support is needed for male partners to help educate HIV-positive mothers’ partners. HIV-positive mothers suggested the need for male mentors to talk to men and encourage them to test for HIV, since it may be difficult for men to speak to a MM.

*“…it is men who understand each other in the way they speak… the result, he knows what his sero-state is. He only denies going to the hospital because he is stubborn; he has, he says, they are lying. So, that requires a man to come and have a conversation until he understands*.” (HIV-positive mother– refused enrollment, 05MR0009).

A key informant and a couple of HCWs suggested increasing the means of transportation due to the weather and long travel distances. One of the HCWs suggested providing protective material against COVID-19. Several MMs asked for folders, pens, notebooks, pens, and a couple requested that the folders be water-resistant and able to withstand wear and tear. One request was made for raincoats as well.

All study participant groups recommended recruiting more MMs. An HIV-positive mother said increasing the number of MMs can help improve awareness. While a few HCWs said recruiting MMs will reduce workload, improve the support provided to HIV-positive mothers living in large districts and give MMs the opportunity to take some annual leave. Moreover, a key informant suggested recruiting more MMs to reach HIV-positive mothers in districts located far from the health facility.

*I feel that we need more mentor mothers. We have peripheries that are very distant from the health unit, that the mentor mothers cannot reach, but that we have mothers there, who need our support…Yes, we feel that the number [of MMs] we have is still… it is little.* (Key-Informant, 09PF0015).

## Discussion

### Theme 1: acceptability of the mentor mother program

The MMP came to be generally well accepted among HIV-positive mothers, MMs and the HCWs. The increased understanding about ART among HIV-positive mothers, positive and supportive relationships between MMs and HIV-positive mothers, support through disclosure and attending the health facility, job satisfaction among MMs, and reduced workload for HCWs all contributed to a general acceptance and appreciation for the MMP.

Through participation I n the MMP, HIV-positive mothers reported gaining a better understanding of why ART was taken daily and the importance of continuing on ART. Previous literature has demonstrated that peer models help HIV-positive mothers understand the risk of HIV transmission during breastfeeding, the importance of disclosing one’s status to at least one person, how to properly administer medication to babies, and the importance of family planning [13, 20]. Improving retention along the PMTCT cascade helps to ensure HIV-exposed infants receive prophylactic ART and helps to increase the rate of mothers following safe infant feeding practices [14, 21, 22].

Previous studies evaluating MM programs have highlighted the strong and trusting relationship between HIV-positive mothers and their MMs. A few studies discussed how MMs’ engagement in attentive listening and making HIV-positive mothers feel like they were ‘on an equal level,’ assisted in reducing self-stigma [23, 24]. Similarly, the Mozambique MMP study had many HIV-positive mothers discuss how they felt more compassion for themselves by having someone to talk to who understood what they were going through. Many HIV-positive mothers reported that their self-stigma was lessened, they felt less isolated and alone, and they felt they had more control over their health status.

MMPs in other locations also reported HIV-positive mothers feeling more comfortable disclosing their HIV status to their families or partners after listening to other HIV-positive mothers tell their disclosure stories during peer-support groups or after forming a close relationship with their peer mentor [25, 26]. Similarly, HIV-positive mothers involved in the Mozambique MMP reported that MMs provided support to help them disclose their HIV-status, including MMs aiding in partner counseling. Disclosing one’s HIV status allowed the mothers to be more involved in PMTCT activities by increasing their ability to attend program activities without fearing that their involvement would lead to the unintended disclosure of their HIV status.

HIV-positive mothers enrolled in other peer-led programs also reported feeling more at ease in the health unit with the presence of MMs because the MMs were viewed as people they could trust [7, 10]. Within the MMP, HIV-positive mothers found it helpful to have MMs attend their health unit appointments with them because they felt their MM could advocate for them with the HCWs.

MMs highly valued their role as MM, discussing at length the strong sense of purpose and satisfaction in helping others. Many MMs and HIV-positive mothers reported appreciating the supportive relationships and community built through the MMP. Previous literature has also identified MM’s devotion to being a MM and enjoyment of their work, also citing the satisfaction of the learning that their patient delivered an HIV-negative baby [27].

Most HCWs participating in the MMP felt their workload was significantly reduced with the support of MMs and they valued the MM’s ability to bring HIV-positive mothers back to the health unit. Previous literature has reported that HCWS have a high workload and less time for patient care and follow-up, making peer-support interventions critical in decreasing cases of lost-to-follow-up [10, 14, 22].

### Theme 2: implementations challenges with the MM program

This study identified many implementation challenges that influenced the initial acceptability of the MMP. The challenges included lack of clarity of the MM roles among HCWs, confidentiality concerns among HIV-positive mothers, MMs need for more clarity regarding their roles and ongoing support for their work, insufficient compensation, and challenges with male partners of HIV-positive mothers.

One of the primary challenges with the MMP was the initial lack of clarity around the MM roles. At the onset of the MMP, HCWs did not understand the role of the MMs, MMs were excluded from health facility meetings and were treated poorly by HCWs. A study in Nigeria reported many challenges between HCWs and MMs due to the non-formal work status, unclear scope of work at the facility level and assignment of non-relevant tasks [27]. The Nigeria study partly attributed the challenge between HCWs and MMs as due to HCW concerns about MM legitimacy and training. This study went on to note that because MM recruitment and engagement process lies outside the formal health sector (often supported by foreign funded donor programs), it is not always recognized as a valid program by government employed HCWs. Other studies have discussed formalizing the MM structure within national health systems in order to provide a sense of continuity and stable income for MMs [28].

Another initial challenge with the MMP was HIV-positive mothers not wanting to receive MMs in their homes due to confidentiality concerns. Some HIV-positive mothers felt apprehensive about receiving home visits, since the MMs were identifiable in the community. These anxieties were further aggravated by the lack of knowledge of the MMs’ confidentiality practices. Findings from other peer programs have highlighted overall acceptance of MM support, while also echoing concerns about privacy and confidentiality due to fear of HIV-status disclosure and consequent stigma and discrimination [3, 26, 29]. The lack of knowledge around confidentiality may have resulted in HIV-positive mothers being hesitant to provide their true addresses to the MMs, resulting in MMs struggling to locate some of the mothers. False addresses and challenges locating the women in the community were not reported in any of the other MM studies reviewed.

Another challenge that affected the program’s success was the MM’s own lack of clarity around their roles. Some MMs reported being unsure of their roles, how to handle certain situations and generally needing more ongoing support than was provided. Previous research has discussed the challenges of MMs having an unclear scope of work and how it may limit their impact [25].

MMs struggled with insufficient compensation and discussed at length having insufficient stipends to do their job, not enough phone credits to call patients and having to use their own resources. Ensuring adequate compensation is critical for the success of peer-support programs, including increased salary or stipend [3, 27, 30].

Many MMs participating in the Mozambique MMP reported male partners being a challenge for MMP implementation. In other peer-led programs, many women reported not being able to take their ART medication properly out of fear of their partner finding the medication and, in turn, knowing their serostatus. This concern has also affected many HIV-positive mothers’ ability to attend health unit visits [25]. To help mitigate this issue, during the home visits, the MMs sometimes utilized male mentors from the Male Champions program at the health facility to help educate and support male-partners of the HIV-positive mothers.

### Theme 3: opportunities to improve the MM program

Study participants shared many recommendations on how to strengthen the MMP and support the development of new MMPs. The main recommendations included greater clarity regarding the roles of MMs, lengthening the MM training and providing refresher training opportunities, ensuring that MMs are not easily identifiable in the community, and providing adequate compensation and resources to the MMs.

Ensuring that the role of the MMP is clear to HIV-positive mothers and HCWs would avoid many of the initial challenges that MMs faced. In order to alleviate fears of unintended HIV status disclosure study participants recommended providing additional information to HIV-positive mothers, specifically explaining the confidentiality practices when visiting homes. HCWs also need to be informed of the role of MM’s in the community and at the facility, and understand the benefits of the MMP. A multi-country study reported that peer mother support recognition and acceptance will be maximized if their role within the health systems is better defined, before and during implementation [31].

Within the Mozambique MMP, there were concerns of MMP staff not retaining previous training. Key informants suggested that all staff attend the new recruits’ trainings since they could serve as refresher courses and boost staff confidence. This suggestion was supported by the HCWs, who suggested that MM trainings be extended beyond five days. Similarly, additional training and refresher courses were successful in the Eswatini peer-support mother program [32]. Previous literature has also recommended providing a training certificate, to legitimize the role of the MM [31].

Future MMP programs should also ensure that MMs are not identifiable with uniforms or any other indicators (such as bicycles or folders) that they are coming from the health facility. A study in Kenya recommended the use of a small identification card instead of uniforms or printed T-shirts [14].

Acknowledging the work that MMs do within these programs and providing them with adequate resources and compensation will promote program sustainability and strengthen MM support, satisfaction, and performance. Ensuring adequate resources may require moving the MMP into the national health care system. One recommendation is to absorb MMs as routine facility staff, provide MMs with more stable employment, and ideally give the MMP more credibility and respect when establishing a MMP in other facilities [31].

The challenge of false addresses suggests that MMs and their patients are not always able to engage in candid discussions about whether patients accept home visits. In addition to building support for the MMP, the MMP in Gaza would benefit from clear training in communicating choice to potential patients and ensuring patients that they may opt out of visits until they are ready to accept a home visit by a mentor mother. Ideally such communication would leave the door open for facility-based adherence support and ongoing discussion of the patient’s willingness to accept a home visit.

### Study strengths and limitations

A significant strength of the study is that data were gathered from many different groups, providing the opportunity to triangulate data and share a more comprehensive understanding of the acceptability and challenges with the MMP. We hope that the data from this study can be helpful when expanding the MMP or developing other MMPs.

A limitation of this study is that women who started the MMP and dropped out and MM’s who resigned were not included in the study population due to limited funding and resources. While these populations were small, they may have offered additional perspectives about the MMP, including additional challenges in receiving and providing services. To address this limitation, questions were included in the data collection tools specifically asking about the challenges that HIV-positive mothers experienced in the MMP and challenges MM’s faced in their role.

## Conclusions

This study highlights that the MMP in Gaza Province provided critical support to HIV-positive mothers in Gaza, resulting in increased understanding of ART and how to take medication, a sense of community, reduced feelings of loneliness, and decreased pressure on the HCWs at health facilities. The study also highlights many opportunities to strengthen the MMP, including the need to provide additional resources to MMs and increase the number of MMs serving the prescribed patient population in order to decrease the workload of existing MMs. This study builds upon an existing evidence base that peer-support programs can be impactful behavior change interventions. While not necessarily generalizable to all settings, the study provides insight for future MMPs working to strengthen ART adherence for HIV-positive mothers within low to middle income communities in Mozambique, sub-Saharan Africa and beyond.

### Electronic supplementary material

Below is the link to the electronic supplementary material.

Supplementary Material 1

Supplementary Material 2

Supplementary Material 3

Supplementary Material 4

Supplementary Material 5

Supplementary Material 6

Supplementary Material 7

## Data Availability

The datasets used and/or analyzed during the current study available from the corresponding author on reasonable request.

## Abbreviations

EA: evaluation assistant
EGPAF: Elizabeth Glaser Pediatric AIDS Foundation
FGD: focus group discussion
HCW: health care worker
IDI: in-depth interview
MM: mentor mother
MMP: mentor mother program
PMTCT: prevention of mother-to-child transmission of HIV
