# Supplementary material for: Evaluation of a peer-support, ‘mentor mother’ program in Gaza, Mozambique; a qualitative study
Source: BMC Health Serv Res. 2024 Mar 27;24:382. doi: 10.1186/s12913-024-10833-3 (PMC10976814; doi:10.1186/s12913-024-10833-3)
Supplement: Supplementary file 1 — Supplementary Material 1 [file 12913_2024_10833_MOESM1_ESM.docx]

**APPENDIX A – FOCAL POINT MENTOR MOTHER IN-DEPTH INTERVIEW GUIDE**

**A Qualitative Evaluation of the Mentor Mother Program for HIV-Positive Pregnant and Lactating Women in Gaza Province, Mozambique*, v.2.3 Apr 1^st^ 2020***

**In-Depth Interview Guide for Focal Point Mentor Mother**

*01=Xai-Xai

02=Limpompo and Chongoene

03= Manjakaze

04= Bilene

05=Chokwe

06=Chibuto

07=Guijá

08=Mabalane

**FPMM= Focal Point Mentor Mother

| Date of the IDI | | __ __ / __ __ / __ __ __ __ (dd-mm-yyyy) |
| --- | --- | --- |
| Study ID | _____/_____/__________ (*Site Number/ **Type of Participant/ IDI Number) | |
| District  Evaluation Assistant Name | | __________________________________ |
| Start time | | __ __ : __ __ |
| End time | | __ __ : __ __ |

**Introduction:**

Introduce yourself as the Evaluation Assistant. Explain that you are here to learn more about the Mentor Mother (MM) Program the Mentor Mother focal point experience with the Program, their opinions about what is working, and any suggestions on how the program can be improved. Remind the participant that there are no right or wrong answers.

| **Section A – demographic Information** |
| --- |

1. Age: __ __ (completed years)
2. Level of education

No school  (1)

Some primary  (2)

Completed primary  (3)

Some secondary  (4)

Completed secondary  (5)

Some degree  (6)

Completed degree  (7)

1. Length of time in this position

__ __ (months) ___ ____ (years

1. Were you a Mentor Mother before

Yes  (1)

No  (2)

1. If yes, for how long?

__ __ (months) ___ ____ (years

| **Section B – Overview of the MM Program** |
| --- |

1. What do you think about the approach of supporting mothers and children through the MM Program?
2. What are some of the benefits of the MM Program on maternal and child care for HIV-positive women and their infants?

3. What are some of the disadvantages of the MM program on maternal and child care for HIV-positive women and their infants?

| **Section C – Experiences of being a Focal Point Mentor Mother** |
| --- |

1. On average, how long have you worked in your HF as a FPMM?
2. How do you feel about the FPMM position being based in health facility?
   Probe: What are the benefits and disadvantages?
3. How do you feel about the amount of guidance and support that you receive in your role as a FPMM?

Probe: Do you feel that you have a support system to rely on? What additional district-level support do you need?

1. What are the challenges you have experienced in your role as a FPMM?

Probe: What are the challenges you experience supporting the mentor mothers?

1. What solutions have you implemented to overcome these problems?
2. How can the role of the FPMM be improved?

| **Section D – HCWs Attitudes towards the MM Program** |
| --- |

1. What is said by HCWs in the HFs (MCH or other sectors) about the Mentor Mother Program?
2. What are your colleagues’ attitudes toward the FPMM presence in the MCH sector?
3. How well is the FPMM received and supported in the HFs?

| **Section E – General Recommendations** |
| --- |

1. What were the challenges for this program implementation (from the initial field installation process until now)?
2. If you could improve this program, what changes would you make?
3. We have reached the end of our interview. Do you have something to add related to anything that we have been talking about?

Thank you for your time!
