# Supplementary material for: Evaluation of a peer-support, ‘mentor mother’ program in Gaza, Mozambique; a qualitative study
Source: BMC Health Serv Res. 2024 Mar 27;24:382. doi: 10.1186/s12913-024-10833-3 (PMC10976814; doi:10.1186/s12913-024-10833-3)
Supplement: Supplementary file 2 — Supplementary Material 2 [file 12913_2024_10833_MOESM2_ESM.docx]

**APPENDIX B – HEALTH CARE WORKERS IN-DEPTH INTERVIEW GUIDE**

**A Qualitative Evaluation of the Mentor Mother Program for HIV-Positive Pregnant and Lactating Women in Gaza Province, Mozambique*, v*.** **2.3 Apr 1^st^, 2020**

**In-Depth Interview Guide for Health Care Workers**

*01=Xai-Xai City

02=Limpompo and Chongoene

03= Manjakaze

04= Bilene

05=Chokwe

06=Chibuto

07=Guijá

08=Mabalane

**HCW= Health Care Workers

| Date of the IDI | | __ __ / __ __ / __ __ __ __ (dd-mm-yyyy) |
| --- | --- | --- |
| Study ID | _____/_____/__________ (*Site Number/ **Type of Participant / IDI Number) | |
| District  Evaluation Assistant Name | | __________________________________ |
| Start time | | __ __ : __ __ |
| End time | | __ __ : __ __ |

|  |  |
| --- | --- |
|  |  |
|  |  |
|  |  |
|  |  |

**Introduction:**

Introduce yourself as the Evaluation Assistant. Explain that you are here to learn more about the Mentor Mothers (MM) Program, Health Professional’s experience as care provider, their opinions about what is working and any suggestions on how the Program can be improved. Remind the participant that there are no right or wrong answers.

| **Section A – demographic Information** |
| --- |

1. Gender

Male  (1)

Female  (2)

1. Age: __ __ (completed years)
2. Department in the health facility

MCH  (1)

Pediatrics  (2)

Community heath  (3)

Pharmacy  (4)

HIV Program  (5)

Other  (6)

Specify__________________________________

1. Type of Health Care Worker

Elementary nurse  (1)

Mid-level nurse  (2)

Preventive Technician  (3)

MCH nurse  (4)

Doctor  (5)

Other  (6)

Specify__________________________________

1. Length of time in this position

__ __ (months) ___ ____ (years

| **Section B – Overview of the MM Program** |
| --- |

1. What do you think about the approach of supporting mothers and children through the MM Program?
2. What are some of the benefits of the MM Program on maternal and child care for HIV-positive women and their infants?
3. What are some of the disadvantages of the MM Program on maternal and child care for HIV-positive women and their infants?
4. What are some of the challenges with the MM Program that you have witnessed?

| **Section C – Individual experiences with the MM Program** |
| --- |

1. On average, how long have you worked with the MM Program in your health facility (HF)?
2. Please describe how your work overlaps with the MM Program.
3. How does the MM Program directly affect your work?
4. How does the MM Program contribute to your department in the HF?

| **Section D – HCWs Attitudes towards the MM Program** |
| --- |

1. What is said by other HCWs in the HF (Mother and Child Health or other sectors) about the MM Program?
2. What are your colleagues’ attitudes toward the FPMM presence in the Mother and Child Health sector?
3. Did you feel that the FPMM is welcomed and supported in the HF? Why or why not?

Please describe any positive or negative aspects of the FPMM.

1. How are the MMs treated by the facility staff?
   Probe: Please describe any positive or negative ways that the MMs are treated by the HCWs.

| **Section E – Challenges and Recommendations** |
| --- |

1. What have been the challenges implementing the MM Program (from the initial field installation process until now)?
2. How have these challenges been addressed?
3. If you could improve this program, what changes would you make?
4. We have reached the end of our interview. Do you have something to add related to anything that we have been talking about?

Thank you for your time!
