# Supplementary material for: Evaluation of a peer-support, ‘mentor mother’ program in Gaza, Mozambique; a qualitative study
Source: BMC Health Serv Res. 2024 Mar 27;24:382. doi: 10.1186/s12913-024-10833-3 (PMC10976814; doi:10.1186/s12913-024-10833-3)
Supplement: Supplementary file 3 — Supplementary Material 3 [file 12913_2024_10833_MOESM3_ESM.docx]

**APPENDIX C – KEY INFORMANT IN-DEPTH INTERVIEW GUIDE**

**A Qualitative Evaluation of the Mentor Mother Program for HIV-Positive Pregnant and Lactating Women in Gaza Province, Mozambique*, v2.3 Apr 1^st^, 2020***

**In-Depth Interview Guide for Key Informants**

*01=Xai-Xai

02=Limpompo and Chongoene

03= Manjakaze

04= Bilene

05=Chokwe

06=Chibuto

07=Guijá

08=Mabalane

**KI= Key Informant (MM Program Manager; MM Program Coordinator; District Mother Focal Point)

| Date of the IDI | | __ __ / __ __ / __ __ __ __ (dd-mm-yyyy) |
| --- | --- | --- |
| Study ID | _____/_____/__________ (*Site Number/ **Type of Participant/ IDI Number) | |
| District  Evaluation Assistant Name | | __________________________________ |
| Start time | | __ __ : __ __ |
| End time | | __ __ : __ __ |

|  |  |
| --- | --- |
|  |  |

**Introduction:**

Introduce yourself as the Evaluation Assistant. Explain that you are here to learn more about the Mentor Mother (MM) Program, the key informant’s experience with the Mentor Mother Program, their opinions about what is working, and any suggestions on how the Program can be improved. Remind the participant that there are no right or wrong answers.

| **Section A – demographic Information** |
| --- |

1. Gender

Male  (1)

Female  (2)

1. Age: __ __ (completed years)
2. Level of education

Completed secondary  (1)

Some degree  (2)

Completed degree  (3)

1. Type of key informant

MM Program Manager  (1)

MM Program Coordinator  (2)

District MM Focal Point  (3)

1. Length of time in this position

__ __ (months) ___ ____ (years

| **Section B – Overview of the MM Program** |
| --- |

1. What do you think about the approach of supporting mothers and children through the MM Program?
2. How has the MM Program affected the care and treatment for HIV-positive women and their children? Please describe the changes you have seen.
3. What do you view as the main benefits or strength of the MM Program?
4. What do you view as the main challenge for the MM Program?

| **Section C –Experiences as a MM Program Manager/Coordinator** |
| --- |

1. Please describe your role and how you currently support the MM Program?
2. On average, how long have you been supporting the MM Program?
3. Please describe your managerial/coordination duties in Mentor Mothers Program.
   Probe: What kinds of activities do you do on a daily basis?
4. How does the MM Program directly affect your work?
5. What challenges have you experienced in your role as a MM Program Manager/Coordinator?
   Probe: Please describe facility and community-level challenges.
6. How have you overcome these challenges?

| **Section D – MM Program Implementation** |
| --- |

1. What do you think about the overall implementation of the Mentor Mother Program?

Probe: What has been successful? What could be improved?

1. What do you think about the implementation at the health facility of the Mentor Mother Program?
   Probe: Please describe any challenges with the MM Program experienced at the health facility.
2. What do you think about the linkage between Mentor Mothers and Health Professionals at health facility? Please describe both the positive and negative aspects.
3. What do you think about the implementation at the community level of the Mentor Mother Program? Please tell me about what steps were taken to inform the community about the Mentor Mothers program.
4. How well was the Mentor Mother’s Program received within the community?
   Probe: Please describe both the positive and negative responses in the community to the Mentor Mother Program.

| **Section E – General Recommendations** |
| --- |

1. What would you change in the current implementation approach of the Mentor Mothers Program and why?
2. What recommendations would you give about the future implementation of the Mentor Mothers Program?
3. What recommendations would you give about the management and coordination of the Mothers program at district or provincial-level?
4. We have reached the end of our interview. Do you have something to add related to anything that we have been talking about?

Thank you for your time!
