# Supplementary material for: Evaluation of a peer-support, ‘mentor mother’ program in Gaza, Mozambique; a qualitative study
Source: BMC Health Serv Res. 2024 Mar 27;24:382. doi: 10.1186/s12913-024-10833-3 (PMC10976814; doi:10.1186/s12913-024-10833-3)
Supplement: Supplementary file 4 — Supplementary Material 4 [file 12913_2024_10833_MOESM4_ESM.docx]

**APPENDIX D – WOMEN ENROLLED IN MENTOR MOTHER PROGRAM IN-DEPTH INTERVIEW GUIDE**

**A Qualitative Evaluation of the Mentor Mother Program for HIV-Positive Pregnant and Lactating Women in Gaza Province, Mozambique*, v.2.3 Apr 1^st^ 2020***

**In-Depth Interview Guide for Enrolled Women in the MM Program**

| Date of the IDI | | __ __ / __ __ / __ __ __ __ (dd-mm-yyyy) |
| --- | --- | --- |
| Study ID | _____/_____/__________ (*Site Number/ **Type of Participant/ IDI Number) | |
| District  Evaluation Assistant Name | | __________________________________ |
| Start time | | __ __ : __ __ |
| End time | | __ __ : __ __ |

*01=Xai-Xai

02=Limpompo and Chongoene

03= Manjakaze

04= Bilene

05=Chokwe

06=Chibuto

07=Guijá

08=Mabalane

**WE= Women Enrolled

|  |  |
| --- | --- |
|  |  |
|  |  |

**Introduction:**

Introduce yourself as the research assistant. Explain that you are here to learn more about the Mentor Mother Program, the women’s experiences receiving the Mentor Mother visits, their opinions about what is working, and any suggestions on how the program can be improved. Remind the participant that there are no right or wrong answers.

| **Section A – demographic Information** |
| --- |

1. Age: __ __ (completed years)
2. Marital state

Married  (1)

Never married  (2)

Separate  (3)

Divorced  (4)

Widowed  (5)

Living with partner  (6)

1. Level of education

No school  (1)

Some primary  (2)

Completed primary  (3)

Some secondary  (4)

Completed secondary  (5)

Some degree  (6)

Completed degree  (7)

1. How long have you known your HIV status?

__ __ (months) ___ ____ (years)

1. Have you disclosed your HIV status to anyone your house?

Yes  (1)

No  (2)

1. How long have you been in antiretroviral treatment?

Started ART during this most recent pregnancy  (1)

Started ART before this most recent pregnancy  (2)

| **Section B – Women’s Reactions to Participate in the Mentor Mothers Program Invitation** |
| --- |

1. What did you initially think when you were first invited to receive MM visits at home?
   Probe: Were you excited about this idea? Did you have any concerns? Were your concerns addressed?
2. What do you think about the process of how you learned about the Mentor Mother Program?
   Probe: What other information would you have liked to have received about the program? How could the program been better explained?
3. Did you accept the offer to receive mentor mothers visits immediately or did you take a few days to think about it?
4. What were the factors that influenced your decision to receive the MM visits?
   Probe: Was anyone involved in your decision to accept the mentor mothers into your home?

| **Section C – General Opinion about the MM Program** |
| --- |

1. What had you heard in your community about the Mentor Mother Program?

1. What are the attitudes in the community towards the services provided by the MMs?
   Probe: In general, is your community accepting and encouraging of the program or does there tend to be more negative perceptions about the program? ask
2. What are the attitudes in your home (among your family) toward the services provided by MMs?
   Probe: Does your family tend to have more negative or positive attitudes towards the MM visits?

| **Section D – Experiences with the MM Program** |
| --- |

1. How did you feel when you first started to receive the MM visits?
2. What messages did the MMs share during their visits to your home?
   Probe: What additional information would have be helpful?
3. Do you feel that you can trust your MM?
   Probe: Do you have any concerns about confidentiality in regards to the MMs? If so, what are your concerns?
4. How has your participation in the MM Program affected your adherence to ART?
   Probe: Overall, do you think the MM visits have improved or worsened your adherence to ART?
5. How has your participation in the MM Program affected your decision to remain in care and attend your health facility?
   Probe: In general, has the MM Program encouraged or discouraged you to return to the health facility for your routine visits?
6. What aspects do you enjoy most about the MM Program?
   Probe: What aspects of the program have the greatest impact on your health?
7. What are some of the challenges you have experienced receiving the MMs at your house?

Probe: Have you had any issues regarding stigma or disclosure as a result of the MMs visiting your home?

1. How did you overcome these challenges?
   Probe: What actions did you specifically take to address the issues you faced?
2. How have your feelings towards the program changed since you first began receiving the MM services?
   Probe: Compared to when you first received the MM visits, are you more or less comfortable now? Has your opinion of the program changed, and, if so, how?
3. In your opinion, how could this program be improved?
   Probe: What recommendations do you have for the program?
4. Would you recommend this program to other women in your community?
5. We have reached the end of our interview. Do you have something to add related to anything that we have been talking about?

Thank you for your time!
