# Supplementary material for: Evaluation of a peer-support, ‘mentor mother’ program in Gaza, Mozambique; a qualitative study
Source: BMC Health Serv Res. 2024 Mar 27;24:382. doi: 10.1186/s12913-024-10833-3 (PMC10976814; doi:10.1186/s12913-024-10833-3)
Supplement: Supplementary file 5 — Supplementary Material 5 [file 12913_2024_10833_MOESM5_ESM.docx]

**APPENDIX E – WOMEN WHO DECLINED THE MENTOR MOTHER PROGRAM VISITS IN-DEPTH INTERVIEW GUIDE**

**Qualitative Evaluation of the Mentor Mother Program for HIV-Positive Pregnant and Lactating Women in Gaza Province, Mozambique*, v. 2.3 Apr 1^st^ 2020***

**In-Depth Interview Guide for Women Who Refused**

*01=Xai-Xai

02=Limpompo and Chongoene

03= Manjakaze

04= Bilene

05=Chokwe

06=Chibuto

07=Guijá

08=Mabalane

**WD= Women Declined

| Date of the IDI | | __ __ / __ __ / __ __ __ __ (dd-mm-yyyy) |
| --- | --- | --- |
| Study ID | _____/_____/__________ (*Site Number/ **Type of Participant / IDI Number) | |
| District  Evaluation Assistant Name | | __________________________________ |
| Start time | | __ __ : __ __ |
| End time | | __ __ : __ __ |

|  |  |
| --- | --- |
|  |  |
|  |  |
|  |  |
|  |  |

**Introduction:**

Introduce yourself as the research assistant. Explain that you are here to learn more about the Mentor Mother Program, about their experience receiving the invitation to be visited by Mentor Mothers, and their opinion about what can be improved in this process. Remind the participant that there are no right or wrong answers.

| **Section A – demographic Information** |
| --- |

1. Age: __ __ (completed years)
2. Level of education

No school  (1)

Some primary  (2)

Completed primary  (3)

Some secondary  (4)

Completed secondary  (5)

Some degree  (6)

Completed degree  (7)

1. Marital state

Married  (1)

Never married  (2)

Separate  (3)

Divorced  (4)

Widowed  (5)

Living with partner  (6)

1. How long have you known your HIV status?

0 – 1 month  (1)

2 – 6 months  (2)

7 – 11 months  (3)

1 year +  (4)

1. Have you disclosed your HIV status to anyone your house?

Yes  (1)

No  (2)

1. How long have you been in antiretroviral treatment?

During pregnant  (1)

Already in treatment  (2)

| **Section B – Overview of Invitation Process** |
| --- |

1. When asked if you would like a Mentor Mother to visit your home, what information was given to you about the program?
2. What do you think about the invitation process of how you learned about the mentor mother Program?

Probe: What other information about the program do you wish had been provided to you upon invitation to have the MMs visit your home?

| **Section C – Women’s Reactions to the Invitations** |
| --- |

1. When you were invited to receive MM at home visits, what were your initial thoughts?
2. Did you refuse the offer to receive MMs immediately or did you take a few days to think about it?

| **Section D – General Opinion about the MM Program** |
| --- |

1. What had you heard in your community about the Mentor Mother Support Program?

1. What are the attitudes in the community towards the services provided by the Mentor Motherss?

Probe: Does the community tend to view the program more negatively or positively? Please explain.

1. What are the general attitudes among families toward the services provided by the Mentor Mothers?
   Probe: Do families tend to have more positive or negative attitudes towards the visits?

| **Section E – Experience with the Invitation to the MM Program** |
| --- |

1. What were the main factors that influenced your decision to decline the Mentor Mother visits?
   Probe: Was anyone else involved in this decision?
2. Were there any other reasons that influenced your decision to not accept the invitation to be visited by the Mentor Mothers?

Probe: Are there any personal reasons that influenced your decision to decline the Mentor Mothers Program, such as concerns with disclosure, knowledge of HIV, knowing others in the program, stigma, etc.?

1. Do you have any fears about receiving the Mentor Mother at your house?

This could include challenges in the community, at home (such as stigma or disclosure), or challenges at the health facility.

1. What would help you to accept the invitation to be visited by the Mentor Mothers?
   Probe for what can encourages women to accept, this could be personal reasons such as disclosure, knowledge of HIV, knowing others Mentor Mother program etc. This could also be from interactions at the facility with the HCW, etc.

| **Section F - Recommendations** |
| --- |

1. Do you have any recommendations on how the Mentor Mother Program can improve its invitation process?
2. Do you have any recommendations on how the Mentor Mother Program can improve their program overall?
3. Do you want to add any additional opinions about the Mentor Mother Program?
4. We have reached the end of our interview. Do you have something to add related to anything that we have been talking about?

Thank you for your time!
