# Supplementary material for: Evaluation of a peer-support, ‘mentor mother’ program in Gaza, Mozambique; a qualitative study
Source: BMC Health Serv Res. 2024 Mar 27;24:382. doi: 10.1186/s12913-024-10833-3 (PMC10976814; doi:10.1186/s12913-024-10833-3)
Supplement: Supplementary file 6 — Supplementary Material 6 [file 12913_2024_10833_MOESM6_ESM.docx]

**APPENDIX F – SUPERVISOR OF MENTOR MOTHER IN-DEPTH INTERVIEW GUIDE**

**A Qualitative Evaluation of the Mentor Mother Program for HIV-Positive Pregnant and Lactating Women in Gaza Province, Mozambique*, v.2.3 Dec 1^st^ 2020***

**In-Depth Interview Guide for Supervisor of Mentor Mother**

*01=Xai-Xai

02=Limpompo and Chongoene

03= Manjakaze

04= Bilene

05=Chokwe

06=Chibuto

07=Guijá

08=Mabalane

**SMM= Supervisor of Mentor Mother

| Date of the IDI | | __ __ / __ __ / __ __ __ __ (dd-mm-yyyy) |
| --- | --- | --- |
| Study ID | _____/_____/__________ (*Site Number/ **Type of Participant / IDI Number) | |
| District  Evaluation Assistant Name | | __________________________________ |
| Start time | | __ __ : __ __ |
| End time | | __ __ : __ __ |

**Introduction:**

Introduce yourself as the Evaluation Assistant. Explain that you are here to learn more about the Mentor Mother (MM) Program, the Supervisor of Mentor Mother’s (SMM) experience with the MM Program, their opinions about what is working, and any suggestions on how the program can be improved. Remind the participant that there are no right or wrong answers.

| **Section A – demographic Information** |
| --- |

1. Age: __ __ (completed years)
2. Level of education

No school  (1)

Some primary  (2)

Completed primary  (3)

Some secondary  (4)

Completed secondary  (5)

Some degree  (6)

Completed degree  (7)

1. How long in this position

__ __ (months) ___ ____ (years

1. Were you a Mentor Mother before

Yes  (1)

No  (2)

1. If yes, for how long?

__ __ (months) ___ ____ (years

| **Section A – Overview the MM Program** |
| --- |

1. What do you think about the approach of supporting mothers and children through the MM Program?
2. What are some of the benefits of the MM Program on maternal and child care for HIV-positive women and their infants?

3. What are some of the disadvantages of the MM Program on maternal and child care for HIV-Positive women and their infants?

| **Section B – Experiences of being a SMM** |
| --- |

1. On average, how long have you worked in your health facility (HF) as a SMM?
2. How do you feel about the SMM position being based in a health facility?
   Probe: What are the benefits and disadvantages of being based at a health facility?
3. How do you feel about the amount of guidance and support that you receive in your role as a SMM?
4. Do you feel that you have a support system to rely on? What additional district level support do you need?
5. What challenges have you experienced in your role as a SMM?

Probe: What are the challenges you experience supporting the MMs?

1. What solutions have you implemented to overcome these problems?
2. How can the role of SMM be improved?

| **Section C – HCWs Attitudes towards the MM Program** |
| --- |

1. What is said by HCWs in the HFs (Mother and Child Health or other sectors) about the MM Program?
2. What are your colleagues’ attitudes toward the SMM presence in the Mother and Child Health sector?
3. How well is the SMM received and supported at the HF?

| **Section D – General Recommendations** |
| --- |

1. What were the challenges for this program’s implementation (from the field installation process until now)?
2. If you could improve this program, what changes would you make?
3. We have reached the end of our interview. Do you have something to add related to anything that we have been talking about?

Thank you for your time!
