# Supplementary material for: Evaluation of a peer-support, ‘mentor mother’ program in Gaza, Mozambique; a qualitative study
Source: BMC Health Serv Res. 2024 Mar 27;24:382. doi: 10.1186/s12913-024-10833-3 (PMC10976814; doi:10.1186/s12913-024-10833-3)
Supplement: Supplementary file 7 — Supplementary Material 7 [file 12913_2024_10833_MOESM7_ESM.docx]

**APPENDIX G – MENTOR MOTHERS’ FOCUS GROUP DISCUSSION GUIDE**

**Qualitative Evaluation of the Mentor Mother Program for HIV-Positive Pregnant and Lactating Women in Gaza Province, Mozambique*, 2.3 Apr 1^st^ 2020***

**Focus Group Discussion Guide for Mentor Mothers**

*01=Xai-Xai

02=Limpompo and Chongoene

03= Manjakaze

04= Bilene

05=Chokwe

06=Chibuto

07=Guijá

08=Mabalane

**MM= Mentor Mothers

| Date of the FGD | | __ __ / __ __ / __ __ __ __ (dd-mm-yyyy) |
| --- | --- | --- |
| Study ID | _____/_____/__________ (*Site Number/ **Type of Participant / IDI Number) | |
| District  Moderator Name  Taker of Notes  Number of FGD Participants | | __________________________________  __________________________________  __ __  __ __ |
| Start time | | __ __ : __ __ |
| End time | | __ __ : __ __ |

|  |  |
| --- | --- |
|  |  |
|  |  |

**Introduction:**

Introduce the moderator and note-taker as the Evaluation Assistant. Explain that you are here to learn more about the Mentor Mother Program, the MMs’ experiences with active tracing and providing home based visits, their opinions about what is working, and any suggestions on how the program can be improved. Remind the participants that there are no right or wrong answers.

| **Section A – General Overview** |
| --- |

1. Please describe your role of being a mentor mother or supporting mother. Probe: What services do mentor mothers provide? Please describe an average day for a mentor mother.
2. What are some of the things that you like most about the Mentor Mother Program?
3. How do you feel about the training you received to be a mentor mother?
   Probe: What additional training would mentor mothers benefit from?
4. How well do you understand the counseling messages that are provided to the mothers?
   Probe: Please describe any counseling messages which need greater clarification.
5. How do you feel about the supervision and mentoring support provided to the mentor mothers?
   Probe: Please describe the supervision/mentoring support that you receive. What additional support do you need?

| **Section B – Program Implementation Barriers and Facilitators** |
| --- |

1. Once you arrive in the community, how do you locate the homes of mothers?

Probe:

1. Do you ask other houses where the mothers house?
2. in cases where you have to ask, do people ask what the issue is that you want to deal with?
3. If you are asked, what kind of answer do you often give?
4. How well do the mothers receive the mentor mothers when they arrive at their doors?
   Probe: Please describe if mentor mothers are generally welcomed and any challenges they might experience being received at the homes.
5. Are there any challenges that the mothers have experienced that you do not feel equipped to deal with? If yes, please describe.
6. In terms of work material to provide this support (pen, forms, guides, notebooks, etc.), do you have any challenges? If yes, what challenges do you specifically face?
7. Do you have any challenges with routine data registration? If yes, what challenges do you face?
8. Do you have any challenges with the compensation you receive for your work? If yes, what challenges do you face?
9. How have you resolved these challenges?

Probe: What has helped you to overcome these challenges? What would help you to better handle these issues?

1. Are there any facilitator factors that motivate or contribute to your activities as mentor mother?

Probe in terms of:

1. training; availability of material;
2. families or community reception;
3. data registration;
4. compensation; etc.
5. What do you like most about your work as a mentor mother?
6. What do you like the least about your work as a mentor mother?

| **Section C – Community Reaction** |
| --- |

1. What were the initial communities’ responses when the program was first implemented?
2. What are the community’s current feelings towards this program?
3. Please tell me about any stigma associated with the families who receive your support?
4. What are some of the reasons that a family might decline to participate in the MM Program?

| **Section D – General Recommendations** |
| --- |

1. If you could improve this program, what changes would you make?
   Probe on the following areas: training and preparation, interactions with the facility and visits to the mothers’ homes.
2. We have reached the end of our interview. Do you have something to add related to anything that we have been talking about?

Thank you for your time!
